# 2019 Outstanding Contributions to ISCB awarded to Barb Bryant

**DOI:** 10.12688/f1000research.19218.1

**Published:** 2019-05-23

**Authors:** Diane Kovats, Ron Shamir, Christiana Fogg

**Affiliations:** 1 International Society for Computational Biology, Leesburg, VA, USA; 2 Blavatnik School of Computer Science, Tel Aviv University, Tel Aviv, Israel; 3 Freelance Writer, Kennsington, MD, USA

**Keywords:** ISCB, Award, Barb Bryant

## Abstract

The Outstanding Contributions to the International Society of Computational Biology (ISCB) Award recognizes outstanding service contributions to the Society by any member through exemplary leadership, education, service, or a combination of these three elements. Barbara (Barb) Bryant, Senior Director at Constellation Pharmaceuticals, is the 2019 ISCB winner of the Outstanding Contributions to ISCB Award and will be recognized at the 2019 Joint Intelligent Systems for Molecular Biology/European Conference on Computational Biology (ISMB/ECCB) in Basel, Switzerland on July 21-25, 2019.

## Barb Bryant: finding community through service

Barb Bryant has spent much of her career as a computational biologist working in the pharmaceutical industry, where she has managed and directed a wide array of bioinformatics projects related to cancer diagnostics, clinical biomarker identification, and mechanism of action of small molecule inhibitors. Bryant first became involved with the International Society of Computational Biology (ISCB) by attending conferences like Intelligent Systems for Molecular Biology (ISMB) and engaging in leadership opportunities through ISCB. She has continued to be involved with ISCB because she has benefited and genuinely treasured being a part of this unique community. She said, “I have enjoyed working with colleagues to find ways to support other computational biologists, particularly students and postdocs. It was great to have a shared purpose, in contrast to the somewhat competitive nature you can sometimes find in scientific research. It is gratifying to be able to see progress on community projects such as nurturing the Student Council, encouraging open sharing of data and software, putting on conferences, or developing publishing venues. Above all, I value the friendships that I have developed with others on the Board and Committees.”

**Figure:**
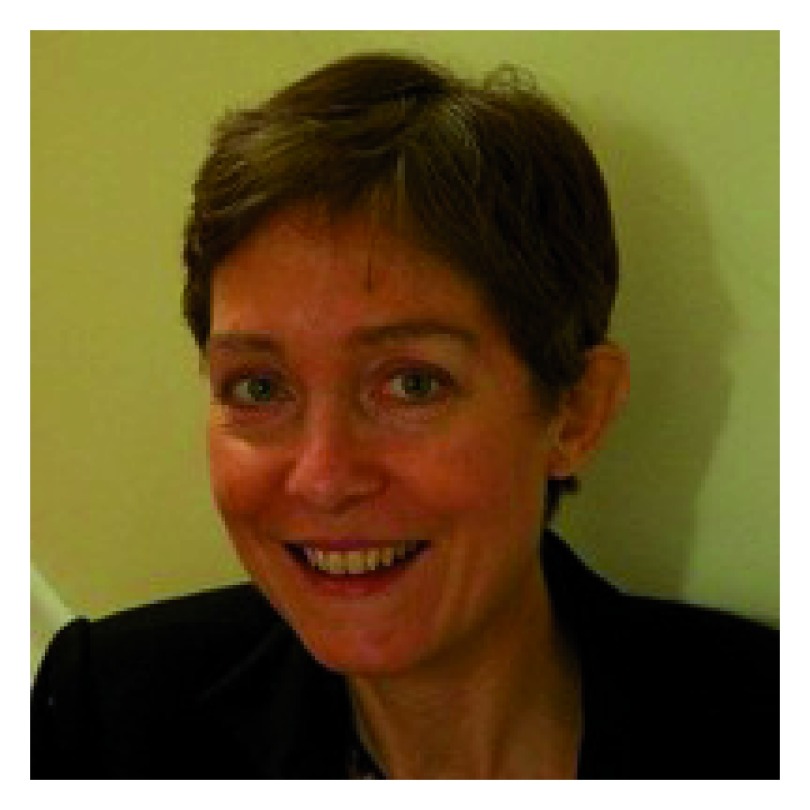
Barb Bryant

Bryant has served on the ISCB Board of Directors in several capacities, including ISCB Secretary (2002–2005) and Vice President (2005–2007). She also chaired the Public Affairs Committee during this time and was instrumental in maintaining ISCB’s affiliation with Federation of American Societies for Experimental Biology (FASEB). Bryant worked on the Editorial Board of *PLoS Computational Biology* and has been thankful for these diverse service opportunities. She said, “I loved collaborating with Phil Bourne on the Editorial Board of *PLoS Computational Biology*. It is great to work with colleagues who have a ton of great ideas and an inclusive, forward-looking attitude. Thinking about how to bring positive change on the Board and within the Society has also been a good challenge. I appreciated serving as the representative of ISCB to FASEB in order to have a voice in Washington at a critical time, post-9/11, when it was becoming harder to travel to the USA for scientific conferences and collaboration.”

Bryant sees ISCB playing a critical future role in advancing important initiatives related to computational biology, including advocating for improved research funding and open access to findings from government funded research. She considers one of ISCB’s strengths to be in the exchange of scientific information through conferences and publications, and she hopes the Society can continue to innovate novel approaches to enhance the communication and dissemination of computational biology research.

Bryant hopes trainees and junior faculty members seek out constructive service opportunities with ISCB and other similar organizations. She said, “There are two key aspects of serving that I think matter even more than the particular area of service. The first is to find a way to make a positive difference -- to change how the world operates. The second is to do it with other people who are positive and effective and fun to be with. If it is a toxic environment, leave. If the people are awesome, stick with it and find a way to contribute, no matter how hard the problem!”

Bryant will be recognized for her distinguished service to ISCB at the 2019 Joint ISMB/European Conference on Computational Biology (ECCB) conference in Basel, Switzerland alongside this year’s other ISCB award recipients.

## Data availability

### Underlying data

No data are associated with this article

